# The evolutionary demise of a social interaction: experimentally induced loss of traits involved in the supply and demand of care

**DOI:** 10.1093/evlett/qrad016

**Published:** 2023-05-02

**Authors:** Eleanor K Bladon, Sonia Pascoal, Nancy Bird, Rahia Mashoodh, Rebecca M Kilner

**Affiliations:** Department of Zoology, University of Cambridge, Cambridge, United Kingdom; Department of Zoology, University of Cambridge, Cambridge, United Kingdom; Department of Haematology, University of Cambridge, Cambridge, United Kingdom; Department of Zoology, University of Cambridge, Cambridge, United Kingdom; Department of Genetics, Evolution and Environment, University College London, London, United Kingdom; Department of Zoology, University of Cambridge, Cambridge, United Kingdom; Department of Genetics, Evolution and Environment, University College London, London, United Kingdom; Department of Zoology, University of Cambridge, Cambridge, United Kingdom

**Keywords:** experimental evolution, trait loss, social evolution, parental care, *Nicrophorus vespilloides*

## Abstract

Phenotypic plasticity enables animals to adjust their behavior flexibly to their social environment—sometimes through the expression of adaptive traits that have not been exhibited for several generations. We investigated how long social adaptations can usefully persist when they are not routinely expressed, by using experimental evolution to document the loss of social traits associated with the supply and demand of parental care. We allowed populations of burying beetles *Nicrophorus vespilloides* to evolve in two different social environments for 48 generations in the lab. In “Full Care” populations, traits associated with the supply and demand of parental care were expressed at every generation, whereas in “No Care” populations we prevented expression of these traits experimentally. We then revived trait expression in the No Care populations at generations 24, 43, and 48 by allowing parents to supply post-hatching care and compared these social traits with those expressed by the Full Care populations. We found that offspring demands for care and male provision of care in the No Care populations were lost sooner than female provision of care. We suggest that this reflects differences in the strength of selection for the expression of alternative traits in offspring, males and females, which can enhance fitness when post-hatching care is disrupted.

## Introduction

Phenotypic plasticity enables animals to flexibly, and rapidly, adjust their behavior according to the environment in which they live—sometimes through the expression of adaptive traits that have not been exhibited for several generations (Davies, 2000; Lahti, 2006). The ability to revive “ghosts of adaptations past” could prove beneficial for populations living in a changing world (Lahti, 2006; Robert & Sorci, 1999; Rothstein, 2001). For this reason, it is important to understand how long such adaptive traits can persist at the population level if they are no longer routinely expressed. This is especially true for behavioral traits that, in comparison with morphological traits (e.g., Waddingham, 1959), have been relatively understudied (Rayner et al., 2022). The evolutionary fate of unexpressed traits is also distinct from recent discussions of the roles of plasticity and genetic assimilation in evolution (Crispo, 2007; DeWitt et al., 1998; Pigliucci et al., 2006; Robinson & Dukas, 1999; Scheiner & Levis, 2021; Snell-Rood et al., 2009), at least according to the definition of genetic assimilation originally proposed by Waddington (1961). Waddington imagined the scenario in which the control of trait expression was moved from environmental induction to genetic control (Crispo 2007). His definition does not encompass the loss of trait expression altogether (since unexpressed traits are not in any sense induced).

The capacity for any unexpressed behavioral trait to persist in the short term could depend on adaptive processes, and whether the original trait is superseded by an existing and more profitable alternative behavior. If there are no net fitness costs associated with plasticity, then the trait might persist—to be vestigially and nonadaptively expressed, occasionally (Rayner et al., 2022). However, selection will act against the mechanisms that enable an unexpressed trait to be environmentally induced if plasticity imposes fitness costs (DeWitt et al., 1998; Snell-Rood et al., 2018), including opportunity costs. In addition, or instead, selection might favor the expression of an existing alternative behavioral trait (e.g., Wright 1961), particularly if this new trait returns greater fitness benefits than the old behavior.

If a trait remains unexpressed in the longer term, then it is not exposed to selection and deleterious mutations can potentially accumulate in the underlying genes (Snell-Rood et al., 2009). Eventually, this will cause the trait to be lost forever (Lahti et al., 2009). Nevertheless, this is likely to happen on a timescale that is beyond experimental work on most animals. Here we are interested instead in the initial stages of trait loss. They have seldom been observed directly, partly because these steps involve relatively subtle nuanced change rather than wholesale disruption (Ellers et al., 2012).

In this study, we used experimental evolution to document the loss of unexpressed behavioral traits in real time. Our work focused on the demand and supply of parental care. It has recently been suggested that social interactions like these are likely to be disrupted by broader environmental change, though the evolutionary implications are still largely unknown (Bailey & Moore, 2018). We mimicked this type of social disruption by experimentally preventing the expression of post-hatching parental care in the burying beetle *Nicrophorus vespilloides* in some populations (“No Care”) for 47 generations, whilst allowing its continued expression in other populations (“Full Care”). After 23, 42, and 47 generations of experimental evolution, we revived interactions between parents and offspring in the No Care populations and compared the expression of the parental and offspring traits involved with the Full Care populations, to measure any changes in the frequency of their expression at the population level and the subsequent fitness effects.

We predicted (a) that we would see less expression of parental and/or larval traits associated with the supply and demand of parental care in No Care populations. We assessed this indirectly with Experiment 1 by measuring brood mass, since it increases with the supply of parental care (Bladon et al., 2020; Pascoal et al., 2018; Steiger, 2013).

Additionally, with Experiments 1 and 2, we quantified the supply of and demand for parental care directly. We predicted (b) that the duration of their care should be shorter in No Care parents than in Full Care parents (Eggert et al., 1998; Experiment 1). We also predicted (c) that No Care larvae should be less inclined to beg from adults (Smiseth and Parker, 2008; Smiseth et al., 2010; Experiment 2).

## Methods

### Natural history of the burying beetle *N. vespilloides*

Burying beetles show elaborate parental care that is highly variable in its duration (Jarrett et al., 2018). A pair converts a dead vertebrate into a nest for their young by tearing off the fur or feathers, covering the flesh with antimicrobial anal and oral exudates, rolling it into a ball and burying it underground (Cotter & Kilner, 2010). The female lays her eggs in the soil around the nest. The larvae crawl to the nest after hatching (Müller & Eggert, 1989). Both parents tend the larvae by defending them from intruders and supplying fluids through oral trophallaxis (Eggert et al., 1998). Parents stay on or very close to the carrion whilst supplying care. Persistent activity away from the carrion indicates that parents have ceased to care for their offspring (de Gasperin et al., 2015). Larvae also self-feed (Smiseth et al., 2003) and can survive (at least in the laboratory) without any post-hatching care. Larval mass is a key correlate of fitness because larval mass predicts adult size (Jarrett et al., 2017), which, in turn, predicts fecundity in both males and females (Bladon et al., 2020; Pascoal et al., 2018).

### Burying beetle husbandry in the laboratory

For all breeding experiments, each pair of sexually mature male and female beetles was bred by placing them in a plastic breeding box (17 × 12 × 6 cm) with damp soil (John Innes Compost) and a 10- to 15-g mouse carcass on which to breed. Larvae were counted and weighed 8 days after pairing and placed in plastic pupation boxes (10 × 10 × 2 cm), filled with damp peat. Sexually immature adults were eclosed approximately 21 days later and housed in an individual box (12 × 8 × 2 cm). Adults were fed twice a week with beef mince until breeding, which took place 15 days post-eclosion. Adults and pupating larvae were kept on a 16L:8D h light cycle at 21°C.

### Experimental evolution

The *N. vespilloides* populations described in this study were part of a long-term experimental evolution project that investigated how populations of burying beetles adapt to the loss of parental care. This project comprised four experimental populations: Full Care (FC; ×2 replicates) and No Care (NC; ×2 replicates). Their establishment and husbandry have been described in detail before (Duarte et al., 2021; Jarrett, Evans, et al., 2018; Jarrett, Rebar, et al., 2018; Rebar et al., 2020; Schrader et al., 2015a, 2015b, 2017). Briefly, these populations were established in 2014 with wild-caught beetles (trapped under permit) from four woodland sites across Cambridgeshire, UK (Byron’s Pool, Gamlingay Woods, Waresley Woods, and Overhall Grove). The NC populations were routinely prevented from supplying any post-hatching care, through the removal of adults at 53 h post-pairing, when the carrion nest was complete but before the larvae had hatched. In the FC populations, adults were allowed to stay with their larvae throughout development and provide care. This procedure was repeated at every generation. Each type of experimental population was run in a separate block (FC1/NC1 and FC2/NC2) with breeding staggered between blocks by 7 days. We used these populations to assess interactions between parents and offspring at generations 24, 43, and 48, both directly and indirectly. Indirect measurements involved partitioning each party’s contribution to offspring mass by the end of larval development.

We have previously described the divergent adaptive evolution of these experimental populations in response to the loss of care. Although the No Care populations initially showed higher rates of larval mortality, they swiftly adapted to the lack of post-hatching care (Schrader et al., 2015b). After 23 generations of experimental evolution, larvae had a similar chance of survival and attained a similar mass at dispersal in each type of population (Rebar et al., 2020; Schrader et al., 2017). Over the same time frame, No Care larvae evolved to hatch more synchronously (Jarrett et al., 2017), to have disproportionately larger mandibles (Jarrett, Evans, et al., 2018), and to be more cooperative with their siblings (Jarrett, Rebar, et al., 2018; Rebar et al., 2020). No Care parents evolved to frontload parental care, making a rounder carrion nest more quickly than Full Care parents, which promoted larval survival in the absence of post-hatching care (Duarte et al., 2021).

### Experiment 1: Assessing the duration of care, and partitioning larval and parental contributions to larval mass at dispersal (Predictions 1 and 2)

We examined the propensity for parents to supply care, and thence contribute to larval mass, by testing the evolving populations after 23 and 42 generations of experimental evolution. While they are committed to providing parental care, parents stay in close proximity to the carcass (De Gasperin et al., 2015). This enables them to tend the carrion, nurture offspring through oral trophallaxis, and to be available for carrion and brood defense. We measured the duration of care exhibited by NC and FC parents by breeding them in a box with a plastic partition that allowed parents to leave the brood and terminate their contribution to care at any time after pairing (Supplementary Figure S1; de Gasperin et al., 2015). We measured the duration of care by monitoring when parents left the nest.

Each breeding pair was placed in the larger compartment (Supplementary Figure S1), along with an 8- to 12-g carcass. At 53 h post-pairing, we cross-fostered parents between boxes to create families where parents either remained with their own nest and eggs or were transferred to a foster nest and eggs of the same (control) or different (cross-fostered) type of experimental population (i.e., No Care or Full Care; see Supplementary Figure S2). This design enabled us to partition the contributions of parents versus offspring to brood mass at dispersal. The transfers within experimental populations enabled us to control for any effects due to the cross-fostering procedure itself. Following any translocation of pairs and nests, adults remained in the boxes until either they terminated care, by entering the escape chamber, or the experiment ended (8 days after pairing), whichever came sooner. Thus, in all treatments, parents were able to supply post-hatching care but were able to cease care at a time of their choosing. From breeding to dispersal, we checked the escape chamber every 4 h between 8 a.m. and 8 p.m. (i.e., 64–192 h after pairing in generation 24; 56–192 h after pairing in generation 43) to measure the duration of care. At dispersal (8 days post-pairing), we removed the remaining parents, counted the number of surviving larvae, and weighed the brood.

### Experiment 2: Assessing larval begging behavior (Prediction 3)

We quantified the frequency of larval begging behavior using previously described methods (Smiseth et al., 2010; Smiseth & Parker, 2008). At generation 48, we set up Full Care pairs and No Care pairs of sexually mature, unrelated, and virgin beetles and put each pair in a standard size breeding box (17 × 12 × 6 cm) with 300-mL soil and a 10- to 13-g mouse carcass. At 53 h after pairing, we removed the male and transferred the female and her carrion nest into a new standard-size breeding box (also containing 300-mL soil). We checked the original breeding box for freshly hatched larvae 24 h later. Larvae were pooled by their experimental population of origin and then distributed to create four treatments: (a) Full Care female with 15 Full Care pooled larvae, (b) Full Care female with 15 No Care pooled larvae, (c) No Care female with 15 Full Care pooled larvae, and (d) No Care female with 15 No Care pooled larvae. This design ensured that larvae did not solicit care from their mother of origin. Larvae were placed directly on the focal female’s carrion nest. Females were only included as focal females in the experiment if their original eggs hatched successfully. This was to prevent cannibalism of larvae that appeared prior to their own eggs hatching (Müller & Eggert, 1990; Smiseth & Parker, 2008).

Twenty-four hours after establishing the experimental broods, when the larvae were second instar and had reached peak begging activity (Smiseth et al., 2003), we removed the females from their broods and placed them in labeled containers in a −20°C freezer for 30 min to euthanize them. Meanwhile, all surviving larvae were removed from their brood ball and placed on labeled pieces of damp paper towel for 25 min prior to the start of the experiment to increase the solicitation of care (T Ratz, personal communication). After removal from the freezer, each female was thawed for 5 min and mounted on a pin at the center of a plastic box (11 × 17 × 4.5 cm) lined with a damp paper towel, mimicking the stance of a parent regurgitating food (Mäenpää & Smiseth, 2017).

The focal female’s experimental brood was then added to the container, with individual larvae placed haphazardly within it, and left for 5 min to acclimatize. We used instantaneous scan sampling to detect begging activity (Mäenpää & Smiseth, 2017; Smiseth et al., 2010; Smiseth & Parker, 2008), recording larval activity every minute for a 10-min period. Activity was classified as either (a) associating with the parent (a larva was within one pronotal width of the female) or (b) begging (a larva was rearing up and touching the parent with its legs) or (c) neither.

### Statistical analyses

All statistical tests were conducted in R version 3.6.1 (R Core Team, 2019). Data handling and visualization were carried out using base R and the “tidyverse” suite of R packages (Wickham et al., 2019). A stepwise deletion method using *F* tests, implemented in the base “statistics” package in R, was used to determine the significance of each term and remove non-significant terms sequentially (Crawley, 2007).

#### Experiment 1

##### Prediction 1: Partitioning parent and offspring contributions to brood mass at dispersal

We initially analyzed the data from generations 24 and 43 together, using a linear model with a Gaussian error structure, implemented by base R regression functions. In the first model, we sought to partition the relative contributions of parents and offspring on brood mass at dispersal. The dependent variable was brood mass at dispersal (g), and the predictor variables included in the maximal model were brood size, carcass mass (g), foster parents’ experimental population (No Care or Full Care), larval experimental population (No Care or Full Care), whether the parents had been transferred between carcasses and eggs or not, male duration of care, female duration of care, experimental block (1 or 2), and the interaction between foster parents’ experimental population and larval experimental population. Subsequent models split the data by generation.

##### Prediction 2: Duration of parental care

We used an ANOVA (with the base R “statistics” package) to determine whether there was a significant difference in the duration of male and female care for each generation separately. As there was a significant difference between the sexes within each generation, male and female parents were analyzed separately in subsequent analyses. We analyzed the duration of parental care using semi-parametric Cox’s proportional models for interval censored data (using the “icenReg” R package; Anderson-Bergman, 2017). In the maximal models for male care, we included parental experimental population (No Care or Full Care), focal offspring experimental population of origin (No Care or Full Care), whether the parents had been transferred or not, number of larvae, carcass mass (g), and experimental block as fixed effects. In the maximal models for female care, the same terms were included but male leaving time was also included to determine whether females were more likely to leave earlier once their partner left. The duration of female care was not included in the male model because there were so few instances of females leaving before their partner (*n* = 9/153 in generation 24; 13/214 in generation 43).

#### Experiment 2

##### Prediction 3: Larval begging behavior

To determine whether there were differences in begging duration between larvae originating from the Full Care and No Care experimental populations, we first ran a quasi-binomial generalized linear model with the average proportion of larvae begging during the scans as the response variable. In a second model, we tested for differences in any behavior of larvae interacting with the (dead) parent by combining begging and associating proportions. In both models, the predictor variables included in the maximal model were larval experimental population (No Care or Full Care), foster female’s experimental population (No Care or Full Care), number of surviving larvae in the brood, experimental block, and the interaction between larval experimental population and foster female’s experimental population.

## Results

### Prediction 1: Partitioning parent and offspring contributions to brood mass at dispersal

Brood mass at dispersal differed significantly between generations (linear regression: *F*_1,361_ = 35.263, *p* < .001), but there were no significant interactions between generation and any other variable, including experimental population of origin (Supplementary Table S1, Supplementary Figures S3 and S4). The difference in brood mass between the generations meant that we could not easily compare generations 24 and 43. Therefore, for all subsequent analyses, we split the data set by generation. We present analyses of generation 43 in the main text because it most closely corresponds with the generation when we analyzed larval begging behavior. Analyses of generation 24 are presented in Supplementary Material (Supplementary Tables S2A and S3A, Supplementary Figures S4–S6).

**Figure 1.**
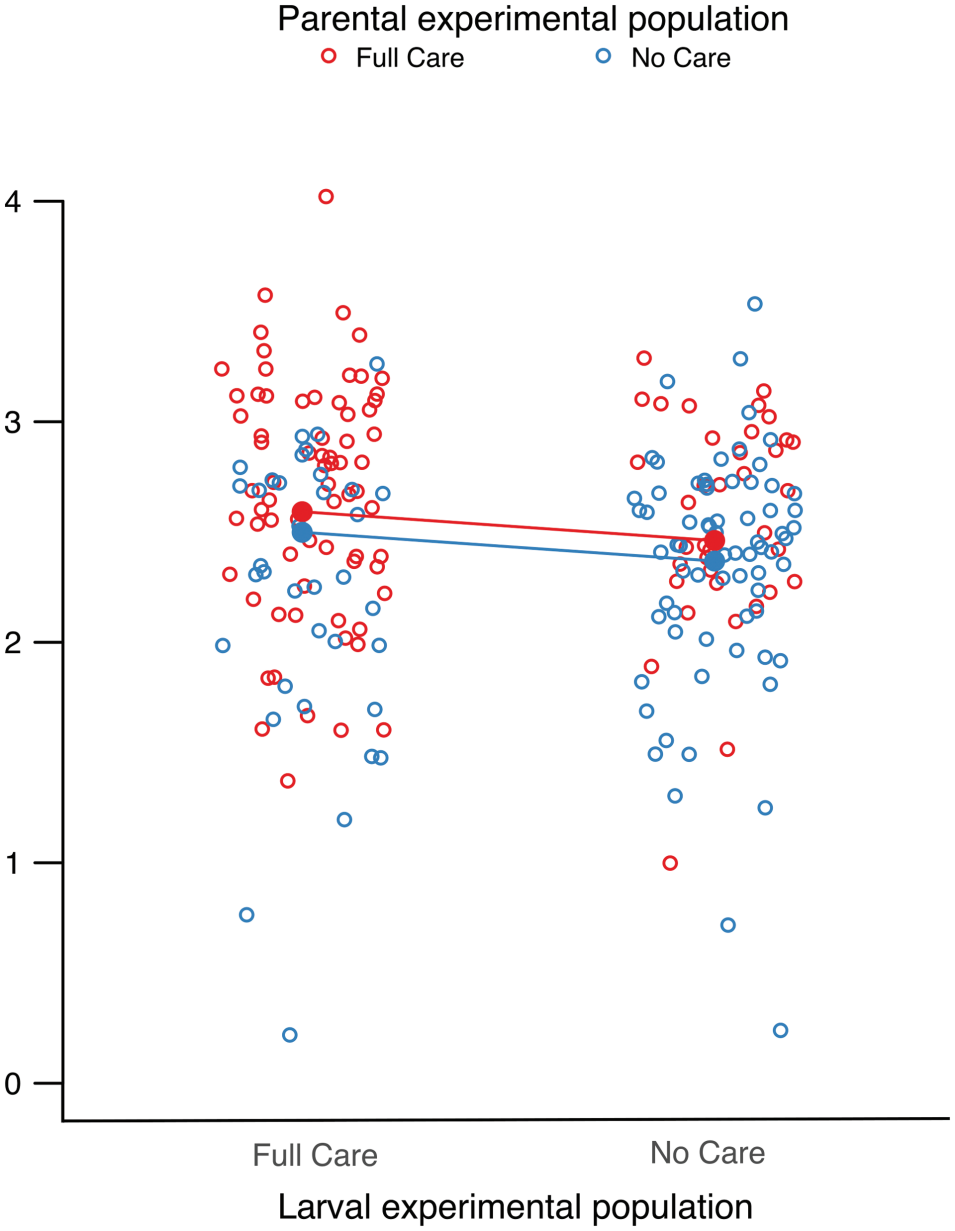
The effect of larval experimental population of origin, and experimental population of current parents, on brood mass after 42 generations of experimental evolution. Solid points represent the predicted means from the minimal model containing both parental experimental population and larval experimental population terms. Open points represent the actual data points—each point corresponds to an individual brood (*n* = 214).

In generation 43, both parental and larval experimental populations contributed to variation in brood mass at dispersal. However, No Care broods of origin attained a lighter mass than Full Care broods of origin (linear regression: *F*_1,208_ = 11.733, *p* = .001, Figure 1), regardless of whether they were raised by Full Care or No Care parents. Conversely, broods raised by No Care parents of origin were lighter than those cared for by Full Care parents of origin (linear regression: *F*_1,208_ = 6.138, *p* = .014), regardless of the larval experimental population of origin (Figure 1). Although the effects of the experimental populations of origin on brood mass at dispersal were small, they were greater than, and independent of, the equivalent effects of carcass and brood size (Supplementary Table S2B).

These results are consistent with Prediction 1. Traits in the No Care offspring prevent them from attaining as great a mass as the Full Care offspring by the time of larval dispersal. Traits in the No Care parents prevent them from enabling larvae in their care to attain as great a mass as larvae cared for by Full Care parents. The next step was to pinpoint whether these traits were connected with, respectively, the demand for and supply of parental care.

### Prediction 2: Duration of parental care

**Figure 2.**
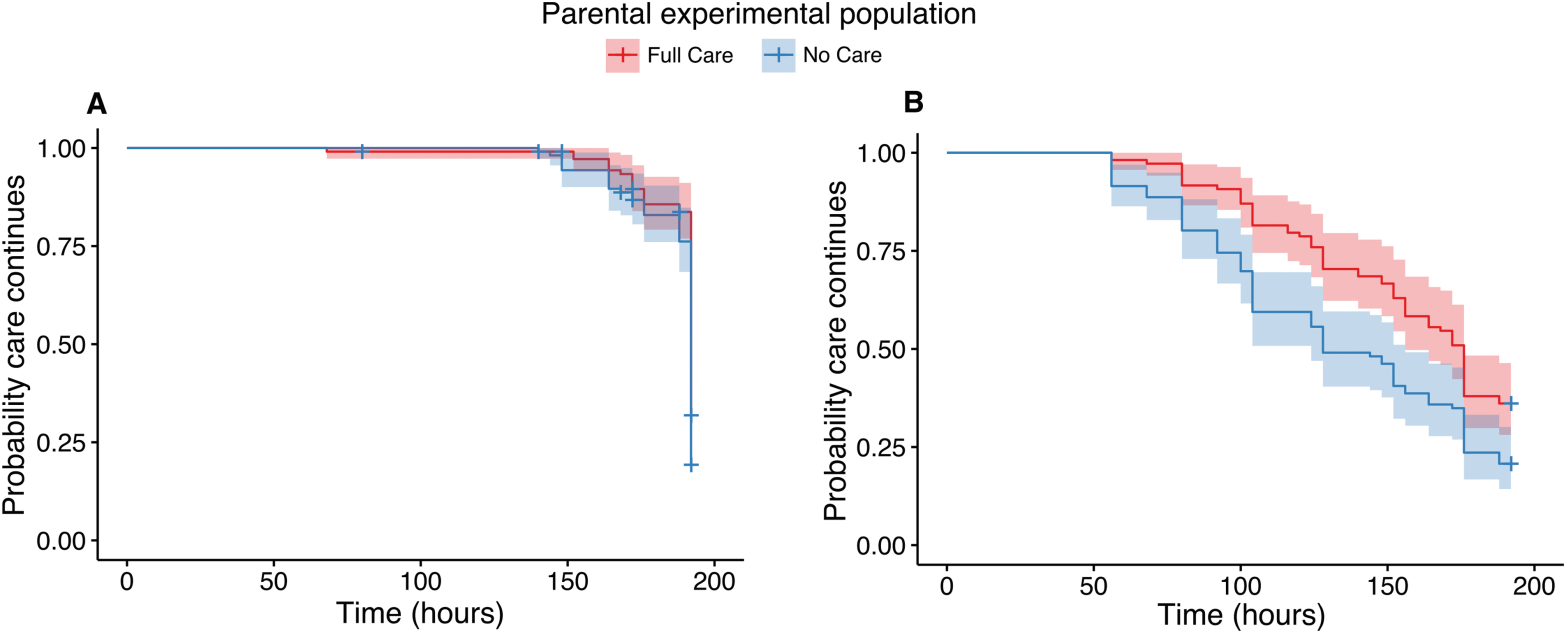
The probability of departing from the nest (with 95% confidence intervals) for (A) female and (B) male parents from No Care (blue) and Full Care (red) experimental populations at generation 43 (Full Care, *n* = 81; No Care, *n* = 72).

Turning first to the supply of care, we found that the duration of maternal care, but not paternal care, significantly contributed to average brood mass at generation 43 (Supplementary Table S3B, female duration of care: linear regression: *F*_1,208_ = 10.477, *p* = .001, male duration of care: linear regression: *F*_1,207_ = 0.004, *p* = .950). No Care males (survival model: hazard ratio = 1.758, Wald = 3.360, *p* < .001, Figure 2B) provided care for significantly less time than Full Care males (Supplementary Table S3B). Although there were no significant effects of experimental population on the duration of female care (Figure 2A, Supplementary Table S3B), female care was generally shorter when their partner provided less prolonged care (survival model: hazard ratio = 0.993, Wald = −2.027, *p* = .021). Fathers left the brood significantly earlier than mothers in both generations (ANOVA: *F*_2,736_ = 185.38, *p* < .001, Supplementary Figure S6). Overall, we find some support for Prediction 2 but only for male care.

### Prediction 3: Larval begging behavior

**Figure 3.**
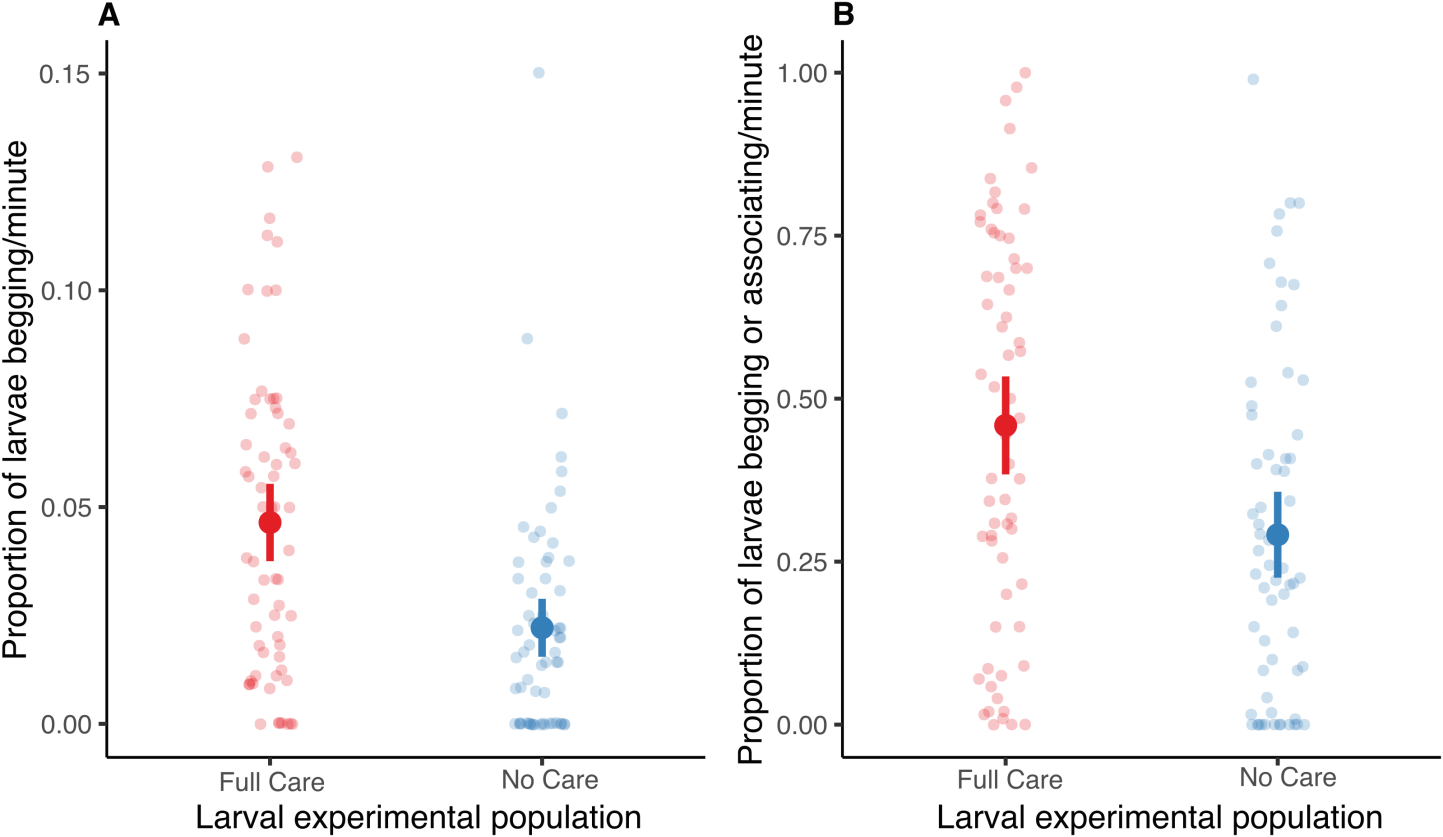
Larval begging intensity in relation to experimental population of origin after 47 generations of experimental evolution. Begging intensity was measured as (A) proportion of larvae begging/minute and (B) proportion of larvae begging or within one pronotal width of the female “foster parent.” The y axis depicts the average of the proportion of larvae displaying the behavior at each sampling scan (once per minute for 10 minutes). Each faint point represents a single brood (*n* = 126). Dark points represent means. Lines represent 95% confidence intervals.

Next, we considered traits in the offspring linked to the demand for care. In support of Prediction 3, a significantly lower proportion of No Care larvae begged toward their foster parent than Full Care larvae during the sampling period (linear regression: *F*_1,124_ = 25.042, *p* < .001, Figure 3A) regardless of the experimental population of their foster parent. The result was similar when considering the total proportion of larvae interacting with the parent (linear regression: *F*_1,124_ = 12.979, *p* = .001, Figure 3B). No other variables significantly affected larval solicitation behaviors (Supplementary Table S4).

## Discussion

We investigated how soon traits connected with offspring demand for care, and parental supply of care, ceased to be expressed after disrupting this social interaction in experimentally evolving populations. We assessed this (a) indirectly, by partitioning the contribution of the offspring and parental experimental population of origin to average brood mass at dispersal, and (b) directly, by quantifying the duration of care supplied by each parent and larval capacity to solicit care. We found some support for all three of the predictions we tested.

Our indirect measurements showed that No Care larvae (on carrion nests prepared by No Care parents) consistently attained a lighter mass at dispersal than Full Care larvae (on carrion nests prepared by Full Care parents), regardless of whether they were raised by parents from the Full Care or No Care experimental populations. We can think of three possible explanations for these findings, which are not mutually exclusive.

First, offspring from the No Care experimental populations may have developed within smaller eggs, meaning that larvae were smaller from hatching. We do not know whether the eggs produced by the No Care populations were smaller after 23 and 42 generations of experimental evolution, but we have previously shown that development in a No Care environment initially yields smaller adults and that smaller females lay smaller eggs (Jarrett et al., 2017).

A second explanation is that the carrion nests produced by No Care parents might have provided an inferior environment for offspring development compared with the carrion nests made by Full Care parents. We reject this explanation because we have demonstrated the opposite to be true (Duarte et al., 2021).

The third explanation is that the demand for care in the No Care experimental populations was lost because it was not expressed in these populations for so many generations. This interpretation is supported by the direct behavioral measurements, which showed that by generation 48, No Care larvae were less likely to solicit care from parents than Full Care larvae. On balance, we conclude that the larval capacity to solicit care started to disappear after generations of not being expressed, and that this loss might have been evident after just 23 generations (although it is possible that egg size also contributed to a lower average brood mass at dispersal in the No Care larvae at this point).

Turning to the parents, by generation 43, there was evidence of decline in No Care males’ supply of post-hatching care, which was now significantly shorter than the care provided by Full Care males. Furthermore, the duration of male care did not predict brood mass at dispersal. In contrast, there was no detectable difference between No Care and Full Care females in the time they spent caring for larvae in generation 43, and the duration of maternal care was still positively associated with average brood mass at this point. Nevertheless, shorter periods of male care curtailed the duration of care supplied by females.

Overall, our experiments show that social behaviors are less likely to be induced when the opportunity to express them has been eliminated for multiple previous generations. Within 48 generations of experimental evolution, larval demand for care and male provision of post-hatching care had degraded. However, we found no evidence that No Care mothers had lost any intrinsic capacity to care for larvae, although the supply of maternal care decreased in response to reduced paternal effort. Comparing patterns of trait loss between larvae, fathers, and mothers reveals differences among members of the family. Offspring have most to lose when this social relationship is disrupted, and least to gain in persisting in demanding care. Mothers have few alternative options for promoting fitness, and most to gain by persisting in supplying care (when possible). By comparison, fathers have more alternative routes to fitness and so gain less by retaining the capacity to supply care.

We suggest that the demand for and the supply of post-hatching care are each traded against the expression of alternative behavioral traits that offer a different route to gaining fitness (Heinen-Kay & Zuk, 2019), with environmental cues determining which trait is more likely to be expressed. The No Care and Full Care environments imposed contrasting selection pressures on the environmental threshold for toggling between alternative behaviors. For example, the expression of larval solicitation behavior probably trades off directly with the expression of self-feeding (Smiseth et al., 2003). In natural populations, from which our experimental populations were founded, there might be standing genetic variation in the environmental cues required to tip the balance between the expression of each larval trait. Selection from the No Care environment favored larvae that were more inclined to express self-feeding at the expense of expressing solicitation behaviors, and recalibrated this trade-off, through genetic accommodation. Hence, after generations of experimental evolution in the No Care environment, re-exposure to a full care environment no longer induced the behaviors that were once induced by this social environment.

Likewise, the expression of male post-hatching care might have changed due to a rebalancing of the trade-off with the supply of prehatching care and, specifically, the effort devoted to making the carrion nest (de Gasperin et al., 2015). Consistent with this suggestion, nest maintenance activity after hatching is known to be negatively genetically correlated with the direct supply of larval care in males (Walling et al., 2008).

Whether an equivalent trade-off exists for females is less clear. The female trait contributing most to fitness before hatching is most likely to be egg size, and it is theoretically possible that there is a negative genetic correlation between egg size and the supply of post-hatching care. However, even if such a correlation exists, it is likely to be concealed by a condition-dependent positive phenotypic correlation between egg size and post-hatching care. Females that receive less post-hatching care are smaller and lay correspondingly smaller eggs (Jarrett et al., 2017).

If trade-offs between competing behavioral routes to fitness explain how some traits are lost, then presumably it is the relative strength of selection to recalibrate the environmentally induced expression of the trade-off that explains why rebalancing happened earlier in larvae and males, but not in females. Larval mortality in the No Care populations was very high in the first few generations of experimental evolution (Schrader et al., 2017). Any larvae that did not self-feed in the No Care environment would rapidly have been selected against. Selection for recalibrating the male’s trade-off arguably is somewhat weaker, since their nest-building behavior less directly affects larval survival. The strength of selection on the putative egg-care trade-off in females could be negligible, especially if its expression is masked by female condition.

The general principle emerging from this study is that behavioral traits cease to be induced environmentally when they become superseded by alternative behavioral traits, which enhance fitness more effectively and which cannot be expressed at the same time as the original behavior. This might be because the alternative behavioral traits are pleiotropic (Wright, 1964) and/or because their simultaneous expression is otherwise physically impossible. This general principle explains the loss of traits associated with parental care in domesticated bird and mammal species because breeders often intervene to nurture offspring themselves or to cross-foster them to other strains that provide better offspring care. As a consequence, wild Norway rats have been shown to be more efficient in pup retrieval than domestic mothers (Price & Belanger, 1977), and some strains of domesticated canary (Vriends, 1992) and zebra finch (Blackwell, 1988) are now incapable of raising offspring to independence.

For behavioral traits like this, a return to the ancestral environment, even after relatively few generations, is not sufficient to restore ancestral levels of trait expression. There are important implications here for conservation captive breeding programs, which could inadvertently induce rapid trait loss through any artificial compensatory husbandry techniques that are introduced to promote breeding success in captivity. Interventions like this could prevent the successful reintroduction of species in the wild by causing the loss of key traits needed for survival and reproduction (Araki et al., 2007; Bowkett, 2009; Williams & Hoffman, 2009).

In general, our study suggests the likelihood of reviving “ghosts of adaptation past” will depend on how long it has been since those adaptive behavioral traits were last expressed and how likely it is that they have been superseded by alternative existing traits that more effectively promote fitness in the new environment. The flexibility of many behavioral traits, and the multiple behavioral routes that are consequently available for achieving similar fitness goals, could mean that some behavioral traits are rapidly substituted and lost following a change in the wider environment.

## Supplementary Material

qrad016_suppl_Supplementary_Material

## Data Availability

The data referred to in this article are openly available on Dryad at doi:10.5061/dryad.r4xgxd2jd.

## References

[CIT0001] Anderson-Bergman, C. (2017). icenReg: Regression models for interval censored data in R. Journal of Statistical Software, 81(12), 1–23. 10.18637/jss.v081.i12

[CIT0002] Araki, H., Cooper, B., & Blouin, M. S. (2007). Genetic effects of captive breeding cause a rapid, cumulative fitness decline in the wild. Science, 318(5847), 100–103. 10.1126/science.1145621 17916734

[CIT0003] Bailey, N. W., & Moore, A. J. (2018). Evolutionary consequences of social isolation. Trends in Ecology & Evolution, 33(8), 595–607. 10.1016/j.tree.2018.05.008 30055910

[CIT0004] Blackwell, C. (1988). Keeping and breeding zebra finches: The complete type standard guide (first). Blandford Press.

[CIT0005] Bladon, E. K., English, S., Pascoal, S., & Kilner, R. M. (2020). Early-life effects on body size in each sex interact to determine reproductive success in the burying beetle *Nicrophorus vespilloides*. Journal of Evolutionary Biology, 33(12), 1725–1734. 10.1111/jeb.13711 33045112

[CIT0006] Bowkett, A. E. (2009). Recent captive-breeding proposals and the return of the ark concept to global species conservation. Conservation Biology, 23(3), 773–776. 10.1111/j.1523-1739.2008.01157.x 19220367

[CIT0007] Cotter, S., & Kilner, R. M. (2010). Sexual division of antibacterial resource defence in breeding burying beetles, *Nicrophorus vespilloides*. Journal of Animal Ecology, 79(1), 35–43. 10.1111/j.1365-2656.2009.01593.x 19627394

[CIT0008] Crawley, M. J. (2007). Multiple regression. In M. J. Crawley (Ed.), The R book (pp. 569–591). John Wiley & Sons, Ltd. 10.1002/9780470515075.ch16

[CIT0009] Crispo, E. (2007). The Baldwin effect and genetic assimilation: Revisiting two mechanisms of evolutionary change mediated by phenotypic plasticity. Evolution, 61(11), 2469–2479. 10.1111/j.1558-5646.2007.00203.x 17714500

[CIT0010] Davies, N. B. (2000). Cuckoos, cowbirds and other cheats (1st edn.). T. & A. D. Poyser, Bloomsbury Collections. 10.5040/9781472597472

[CIT0011] De Gasperin, O., Duarte, A., & Kilner, R. M. (2015). Interspecific interactions explain variation in the duration of paternal care in the burying beetle. Animal Behaviour, 109, 199–207. 10.1016/j.anbehav.2015.08.014 26778845 PMC4686539

[CIT0012] DeWitt, T. J., Sih, A., & Wilson, D. S. (1998). Costs and limits of phenotypic plasticity. Trends in Ecology and Evolution, 13, 77–81. 10.1016/S0169-5347(97)01274-3 21238209

[CIT0013] Duarte, A., Rebar, D., Hallett, A. C., Jarrett, B. J. M., & Kilner, R. M. (2021). Evolutionary change in the construction of the nursery environment when parents are prevented from caring for their young directly. Proceedings of the National Academy of Sciences, 118(48), e2102450118. 10.1073/pnas.2102450118 PMC8640939 34819363

[CIT0014] Eggert, A. -K., Reinking, M., & Müller, J. K. (1998). Parental care improves offspring survival and growth in burying beetles. Animal Behaviour, 55(1), 97–107. 10.1006/anbe.1997.0588 9480676

[CIT0015] Ellers, J., Kiers, E. T., Currie, C. R., McDonald, B. R., & Visser, B. (2012). Ecological interactions drive evolutionary loss of traits. Ecology Letters, 15(10), 1071–1082. 10.1111/j.1461-0248.2012.01830.x 22747703

[CIT0016] Heinen-Kay, J. L., & Zuk, M. (2019). When does sexual signal exploitation lead to signal loss? Frontiers in Ecology and Evolution, 7. 10.3389/fevo.2019.00255

[CIT0017] Jarrett, B. J. M., Evans, E., Haynes, H. B., Leaf, M. R., Rebar, D., Duarte, A., Schrader, M., & Kilner, R. M. (2018). A sustained change in the supply of parental care causes adaptive evolution of offspring morphology. Nature Communications, 9(1), 3987. 10.1038/s41467-018-06513-6 PMC6162320 30266903

[CIT0018] Jarrett, B. J. M., Rebar, D., Haynes, H. B., Leaf, M. R., Halliwell, C., Kemp, R., & Kilner, R. M. (2018). Adaptive evolution of synchronous egg-hatching in compensation for the loss of parental care. Proceedings of the Royal Society B: Biological Sciences, 285(1885), 20181452. 10.1098/rspb.2018.1452 PMC6125895 30158310

[CIT0019] Jarrett, B. J. M., Schrader, M., Rebar, D., Houslay, T. M., & Kilner, R. M. (2017). Cooperative interactions within the family enhance the capacity for evolutionary change in body size. Nature Ecology & Evolution, 1, 0178. 10.1038/s41559-017-0178 28685165 PMC5495167

[CIT0020] Lahti, D. C. (2006). Persistence of egg recognition in the absense of cuckoo brood parasitism: Pattern and mechanism. Evolution, 60(1), 157–168. 10.1111/j.0014-3820.2006.tb01090.x 16568640

[CIT0021] Lahti, D. C., Johnson, N. A., Ajie, B. C., Otto, S. P., Hendry, A. P., Blumstein, D. T., Coss, R. G., Donohue, K., & Foster, S. A. (2009). Relaxed selection in the wild. Trends in Ecology & Evolution, 24(9), 487–496. 10.1016/j.tree.2009.03.010 19500875

[CIT0022] Mäenpää, M. I., & Smiseth, P. T. (2017). Egg size, begging behaviour and offspring fitness in *Nicrophorus vespilloides*. Animal Behaviour, 134, 201–208. 10.1016/j.anbehav.2017.10.014

[CIT0023] Müller, J. K., & Eggert, A-K. (1989). Paternity assurance by “helpful” males: Adaptations to sperm competition in burying beetles. Behavioral Ecology and Sociobiology, 24(4), 245–249. 10.1007/BF00295204

[CIT0024] Müller, J. K., & Eggert, A-K. (1990). Time-dependent shifts between infanticidal and parental behavior in female burying beetles a mechanism of indirect mother-offspring recognition. Behavioral Ecology and Sociobiology, 27(1), 11–16. 10.1007/BF00183307

[CIT0025] Pascoal, S., Jarrett, B., Evans, E., & Kilner, R. (2018). Superior stimulation of female fecundity by subordinate males provides a mechanism for telegony. Evolution Letters, 2(2), 114–125. 10.1002/evl3.45 30283669 PMC6121788

[CIT0026] Pigliucci, M., Murren, C. J., & Schlichting, C. D. (2006). Phenotypic plasticity and evolution by genetic assimilation. Journal of Experimental Biology, 209(12), 2362–2367. 10.1242/jeb.02070 16731812

[CIT0027] Price, E. O., & Belanger, P. L. (1977). Maternal behavior of wild and domestic stocks of Norway rats. Behavioral Biology, 20(1), 60–69. 10.1016/s0091-6773(77)90511-9

[CIT0028] Rayner, J. G., Sturiale, S. L., & Bailey, N. W. (2022). The persistence and evolutionary consequences of vestigial behaviours. Biological Reviews, 97(4), 1389–1407. 10.1111/brv.12847 35218283 PMC9540461

[CIT0029] R Core Team. (2019). R: A language and environment for statistical computing. R Foundation for Statistical Computing. https://www.r-project.org

[CIT0030] Rebar, D., Bailey, N. W., Jarrett, B. J. M., & Kilner, R. M. (2020). An evolutionary switch from sibling rivalry to sibling cooperation, caused by a sustained loss of parental care. Proceedings of the National Academy of Sciences, 117, 2544–2550. 10.1073/pnas.1911677117 PMC7007579 31964847

[CIT0031] Robert, M., & Sorci, G. (1999). Rapid increase of host defence against brood parasites in a recently parasitized area: The case of village weavers in Hispaniola. Proceedings of the Royal Society B: Biological Sciences of the United States of America, 266(1422), 941–946. 10.1098/rspb.1999.0727

[CIT0032] Robinson, B. W., & Dukas, R. (1999). The influence of phenotypic modifications on evolution: The Baldwin effect and modern perspectives. Oikos, 85(3), 582–589. 10.2307/3546709

[CIT0033] Rothstein, S. I. (2001). Relic behaviours, coevolution and the retention versus loss of host defences after episodes of avian brood parasitism. Animal Behaviour, 61(1), 95–107. 10.1006/anbe.2000.1570 11170700

[CIT0034] Scheiner, S. M., & Levis, N. A. (2021). The loss of phenotypic plasticity via natural selection: Genetic assimilation. In Phenotypic plasticity & evolution: Causes, consequences, controversies (1st edn, pp. 161–177). CRC Press. 10.1201/9780429343001

[CIT0035] Schrader, M., Jarrett, B. J. M., & Kilner, R. M. (2015a). Parental care masks a density-dependent shift from cooperation to competition among burying beetle larvae. Evolution, 69(4), 1077–1084. 10.1111/evo.12615 25648525 PMC4476075

[CIT0036] Schrader, M., Jarrett, B. J. M., & Kilner, R. M. (2015b). Using experimental evolution to study adaptations for life within the family. The American Naturalist, 185(5), 610–619. 10.1086/680500 PMC4497813 25905504

[CIT0037] Schrader, M., Jarrett, B. J. M., Rebar, D., & Kilner, R. M. (2017). Adaptation to a novel family environment involves both apparent and cryptic phenotypic changes. Proceedings of the Royal Society B: Biological Sciences, 284(1862), 20171295. 10.1098/rspb.2017.1295 PMC5597835 28878064

[CIT0038] Smiseth, P. T., Andrews, C., Brown, E., & Prentice, P. M. (2010). Chemical stimuli from parents trigger larval begging in burying beetles. Behavioral Ecology, 21(3), 526–531. 10.1093/beheco/arq019

[CIT0039] Smiseth, P. T., Darwell, C. T., & Moore, A. J. (2003). Partial begging: An empirical model for the early evolution of offspring signalling. Proceedings of the Royal Society B: Biological Sciences, 270(1526), 1773–1777. 10.1098/rspb.2003.2444 PMC1691438 12964978

[CIT0040] Smiseth, P. T., & Parker, H. J. (2008). Is there a cost to larval begging in the burying beetle *Nicrophorus vespilloides*? Behavioral Ecology, 19(6), 1111–1115. 10.1093/beheco/arn101

[CIT0041] Snell-Rood, E. C., Kobiela, M. E., Sikkink, Kristin L., & Shephard, A. M. (2018). Mechanisms of plastic rescue in novel environments. Annual Review of Ecology, Evolution, and Systematics, 49(1), 331–354. 10.1146/annurev-ecolsys-110617-062622

[CIT0042] Snell-Rood, E. C., Van Dyken, J. D., Cruickshank, T., Wade, M. J., & Moczek, A. P. (2009). Toward a population genetic framework of developmental evolution: The costs, limits, and consequences of phenotypic plasticity. Bioessays, 32(1), 71–81. 10.1002/bies.200900132 PMC3151734 20020499

[CIT0043] Steiger, S. (2013). Bigger mothers are better mothers: disentangling size-related prenatal and postnatal maternal effects. Proceedings of the Royal Society B: Biological Sciences, 280(1766), 20131225. 10.1098/rspb.2013.1225 PMC3730594 23843390

[CIT0044] Vriends, M. M. (1992). The new canary handbook. Barron’s Educational Series Inc.

[CIT0045] Waddingham, C. H. (1959). Canalization of development and genetic assimilation of acquired characters. Nature, 183(4676), 1654–1655. 10.1038/1831654a0 13666847

[CIT0046] Waddington, C. H. (1961). Genetic assimilation. In E. W. Caspari & J. M. Thoday (Eds.), Advances in genetics (Vol. 10, pp. 257–293). Academic Press. 10.1016/S0065-2660(08)60119-4 14004267

[CIT0047] Walling, C. A., Stamper, C. E., Smiseth, P. T., & Moore, A. J. (2008). The quantitative genetics of sex differences in parenting. Proceedings of the National Academy of Sciences, 105(47). 10.1073/pnas.0803146105 PMC2587554 19008350

[CIT0048] Wickham, H., Averick, M., Bryan, J., Chang, W., D’Agostino McGowan, L., Romain, F., Grolemund, G., Hayes, A., Henry, L., Hester, J., Kuhn, M., Lin Pedersen, T., Miller, E., Milton Bache, S., Müller, K., Ooms, J., Robinson, D., Seidel, D. P., Spinu, V., & Yutani, H. (2019). Welcome to the tidyverse. Journal of Open Source Software, 4(43), 1686. 10.21105/joss.01686

[CIT0049] Williams, S. E., & Hoffman, E. A. (2009). Minimizing genetic adaptation in captive breeding programs: A review. Biological Conservation, 142(11), 2388–2400. 10.1016/j.biocon.2009.05.034

[CIT0050] Wright, S. (1964). Pleiotropy in the evolution of structural reduction and of dominance. The American Naturalist, 98(899), 65–69. 10.1086/282301

