# Supplementary material for: The evolutionary demise of a social interaction: experimentally induced loss of traits involved in the supply and demand of care
Source: Evol Lett. 2023 May 2;7(3):168–75. doi: 10.1093/evlett/qrad016 (PMC10210443; doi:10.1093/evlett/qrad016)
Supplement: qrad016_suppl_Supplementary_Material [file qrad016_suppl_supplementary_material.pdf]

Supplementary Material

Table S1 – Results of a Gaussian linear model analysing predictors of brood mass at dispersal for both generations 24 and 43 combined. Significant terms (retained in the minimal model) are shown in bold. All terms included in the maximal model are given. Statistics are given for the last model in which the term was retained. ":" represents an interaction between terms.

**Independent variable**	**Estimate**	**SE**	***F***	**df**	***p***
Intercept	-0.839	0.208			
Generation (43)	**0.175**	**0.030**	**35.263**	**1**	**<0.001**
Carcass mass (g)	**0.113**	**0.017**	**43.917**	**1**	**<0.001**
Brood size	**0.077**	**0.002**	**1209.700**	**1**	**<0.001**
Current parents' experimental population (No Care)	**-0.097**	**0.028**	**11.905**	**1**	**0.001**
Larval experimental population (No Care)	**-0.127**	**0.028**	**20.37**	**1**	**<0.001**
Transferred? (Yes)	-0.044	0.028	2.517	1	0.114
Male duration of care	<0.001	<0.001	0.206	1	0.100
Female duration of care	**0.003**	**0.001**	**11.258**	**1**	**0.001**
Block (2)	**-0.104**	**0.027**	**15.072**	**1**	**<0.001**
Generation:Current parents' experimental population	-0.059	0.054	1.218	1	0.270
Generation:Larval experimental population	0.592	0.554	0.351	1	0.554
Current parents' experimental population:Larval experimental population	-0.071	0.057	0.057	1	0.812

Table S2 – Results of a Gaussian linear model analysing predictors of brood mass at dispersal for each generation separately. Results are shown for (A) generation 24 and B) generation 43. Significant terms (retained in the minimal model) are shown in bold. All terms included in the maximal model are given. Statistics are given for the last model in which the term was retained. ":" represents an interaction between terms.

**Independent variable**	**Estimate**	**SE**	***F***	**df**	***p***	**Estimate**	**SE**	***F***	**df**	***p***
	**A) Generation 24**					**B) Generation 43**				
Intercept	-0.945	0.268				-0.695	0.307			
Carcass mass (g)	**0.134**	**0.022**	**36.811**	**1**	**<0.001**	**0.102**	**0.023**	**19.661**	**1**	**<0.001**
Brood size	**0.067**	**0.003**	**540.18**	**1**	**<0.001**	**-0.080**	**0.003**	**748.16**	**1**	**<0.001**
Current parents' experimental population (No Care)	-0.049	0.037	1.767	1	0.186	**-0.093**	**0.036**	**6.775**	**1**	**0.010**
Larval experimental population (No Care)	**-0.168**	**0.037**	**20.274**	**1**	**<0.001**	**-0.131**	**0.035**	**13.744**	**1**	**<0.001**
Transferred? (Yes)	0.026	0.038	0.463	1	0.497	**-0.074**	**0.036**	**4.358**	**1**	**0.038**
Male duration of care	**0.001**	**0.001**	**4.921**	**1**	**0.028**	<0.001	<0.001	0.173	1	0.678
Female duration of care	**0.002**	**0.001**	**6.005**	**1**	**0.016**	**0.003**	**0.001**	**8.565**	**1**	**0.004**
Block (2)	**-0.114**	**0.038**	**8.885**	**1**	**0.003**	**-0.070**	**0.033**	**4.488**	**1**	**0.035**
Current parents' experimental population:Larval experimental population	0.059	0.088	0.448	1	0.505	-0.066	0.081	0.659	1	0.418

Table S3 – Results of semi-parametric Cox's proportional models of duration of parental care in A) generation 24 and B) generation 43. Terms retained in the minimal model are shown in bold. All terms included in the maximal model are given. Statistics given are for the last model in which the term was retained ":" represents an interaction between terms.

Parent	Independent Variable	Parameter Estimate	Hazard Ratio	SE	z	*p*	Parameter Estimate	Hazard Ratio	SE	z	*p*
		A) Generation 24					***B) Generation 43***				
Male	Male experimental population (No Care)	0.073	1.076	0.170	0.430	0.680	**0.564**	**1.758**	**0.168**	**3.360**	**<0.001**
	Brood experimental population (No Care)	0.022	1.022	0.218	0.099	0.908	-0.322	0.725	0.177	-1.823	0.062
	Carcass mass (g)	0.011	1.011	0.013	0.081	0.927	0.106	1.112	0.105	1.009	0.317
	Transferred? (Yes)	0.151	1.163	0.169	0.893	0.402	-0.116	0.891	0.167	-0.691	0.505
	Brood size	0.003	1.003	0.013	0.224	0.833	-0.007	0.993	0.017	-0.380	0.659
	Block (1)	0.155	1.168	0.197	0.791	0.382	**0.365**	**1.441**	**0.169**	**2.160**	**0.026**
Female	Female experimental population (No Care)	0.548	1.730	0.284	1.934	0.426	0.082	1.086	0.335	0.246	0.794
	Brood experimental population (No Care)	**0.559**	**1.749**	**0.268**	**2.085**	**0.028**	-0.132	0.876	0.266	-0.497	0.639
	Carcass mass (g)	-0.071	0.932	0.151	-0.469	0.673	-0.170	0.843	0.188	-0.904	0.390
	Transferred? (Yes)	0.020	1.020	0.389	0.052	0.946	0.027	1.027	0.350	0.077	0.929
	Brood size	0.038	1.038	0.022	1.675	0.061	0.024	1.024	0.024	0.986	0.318
	Block (1)	0.254	1.289	0.230	0.849	0.355	0.096	1.100	0.295	0.324	0.736
	Male duration of care	-0.007	0.993	0.004	-1.671	0.057	**-0.007**	**0.993**	**0.004**	**-2.027**	**0.021**

Table S4 – Quasi-binomial GLMs fitted to explain variation in the proportion of larvae begging and the proportion of larvae either begging or associating with the parent at each sampling scan (scanning took place every minute for 10 minutes). "Begging" was defined as occurring when a larva reached up to touch the parent with its forelegs and "associating" was defined as a larva being within a pronotum's width of the parent's body. All terms included in the maximal model are shown, as well as their contribution to the final model. Terms retained in the minimal model are shown in bold. ":" represents an interaction between terms.

**Dependent variable**	**Independent variable**	**Estimate**	**SE**	***F***	**d.f.**	***p***
Begging	**Brood experimental population (No Care)**	**-0.850**	**0.176**	**25.042**	**1**	**<0.001**
	Foster Female's experimental population (No Care)	-0.109	0.163	0.450	1	0.503
	Brood size	-0.041	0.037	1.243	1	0.267
	Block (1)	0.036	0.169	0.046	1	0.830
	Brood experimental population:Foster Female's experimental population	-0.191	0.353	0.294	1	0.589
Interacting with the parent (begging and associating combined)	**Brood experimental population (No Care)**	**-0.788**	**0.221**	**12.979**	**1**	**0.001**
	Foster Female's experimental population (No Care)	0.170	0.222	0.585	1	0.446

	Brood size	0.047	0.053	0.795	1	0.375
	Block (1)	0.319	0.226	2.009	1	0.159
	Brood experimental population:Foster Female's experimental population	0.210	0.449	0.219	1	0.641

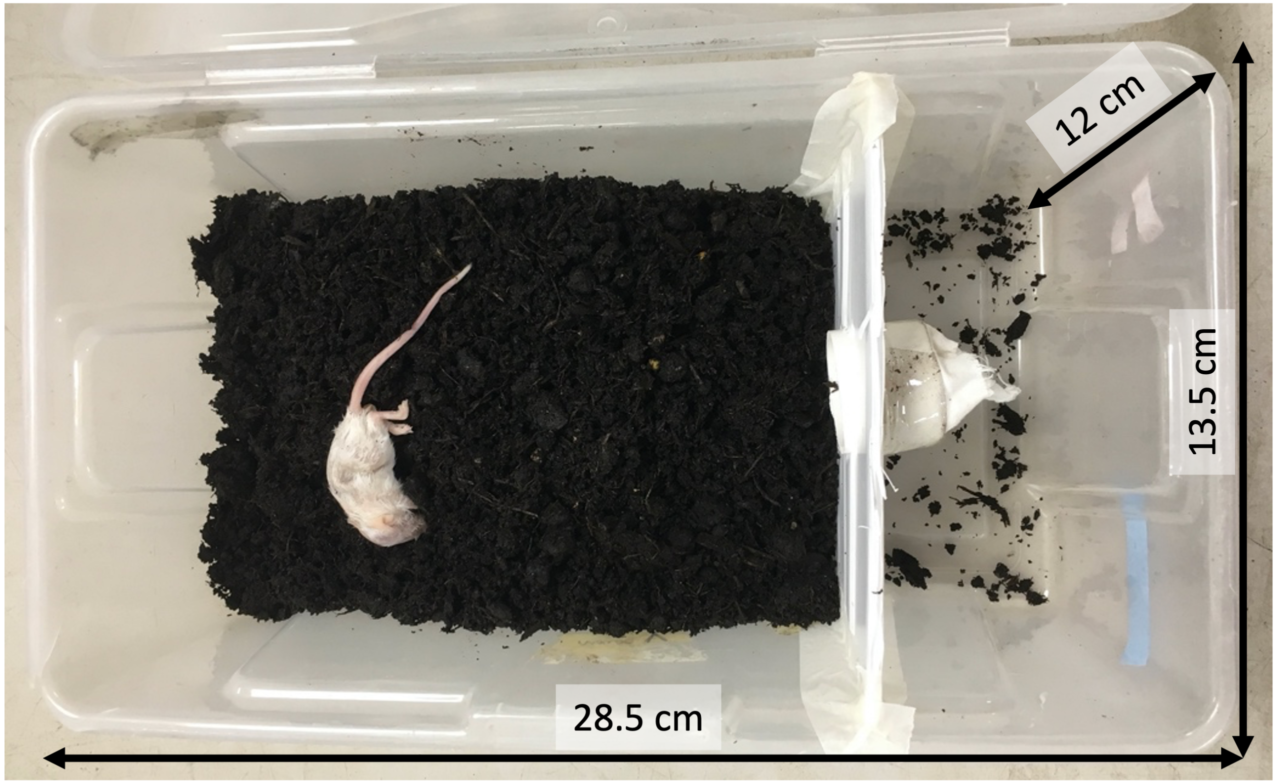

Figure S1 - The breeding boxes used in Experiment 1 with breeding compartment (left) and "escape chamber" (right). The two compartments are separated by a partition with a one-way valve, that allowed adult beetles to move from the breeding compartment to the escape chamber but not back again.

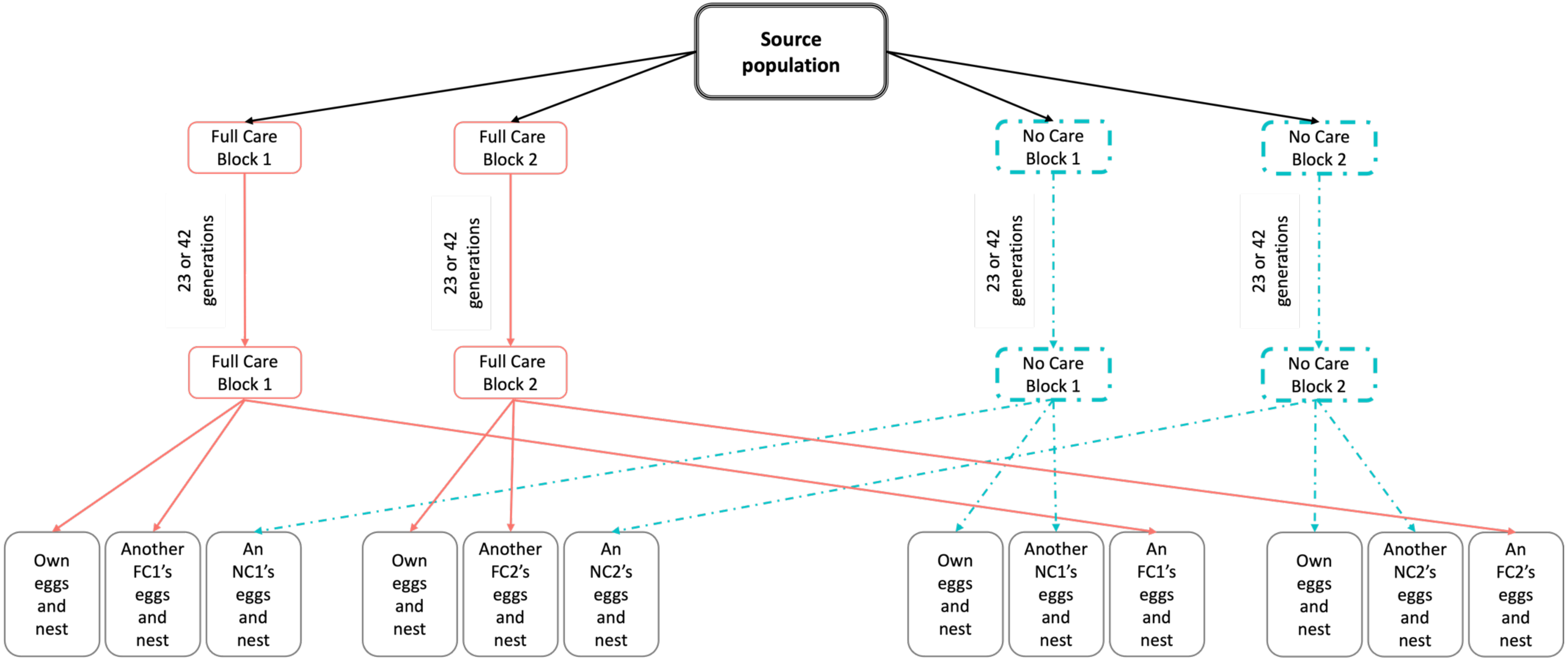

Figure S2 – Overview of experimental evolution, showing how the different evolving populations were used in Experiment 1.

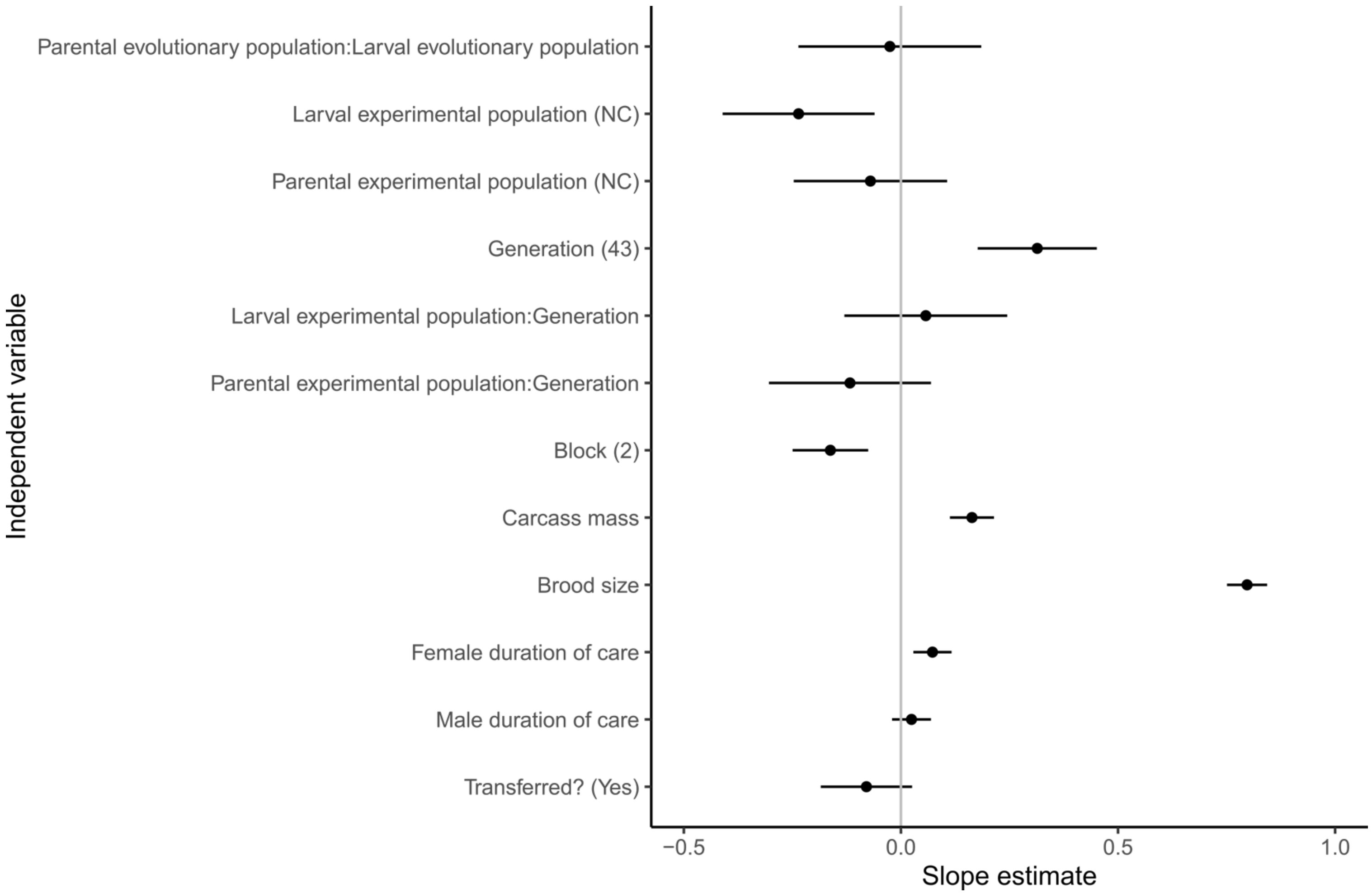

Figure S3 – Coefficient plot showing the value of the standardised regression coefficient (points) with 95% confidence intervals (horizontal lines) for each independent variable, and including both generations (analysis reported in Table S1). All variables in the maximal model are shown.

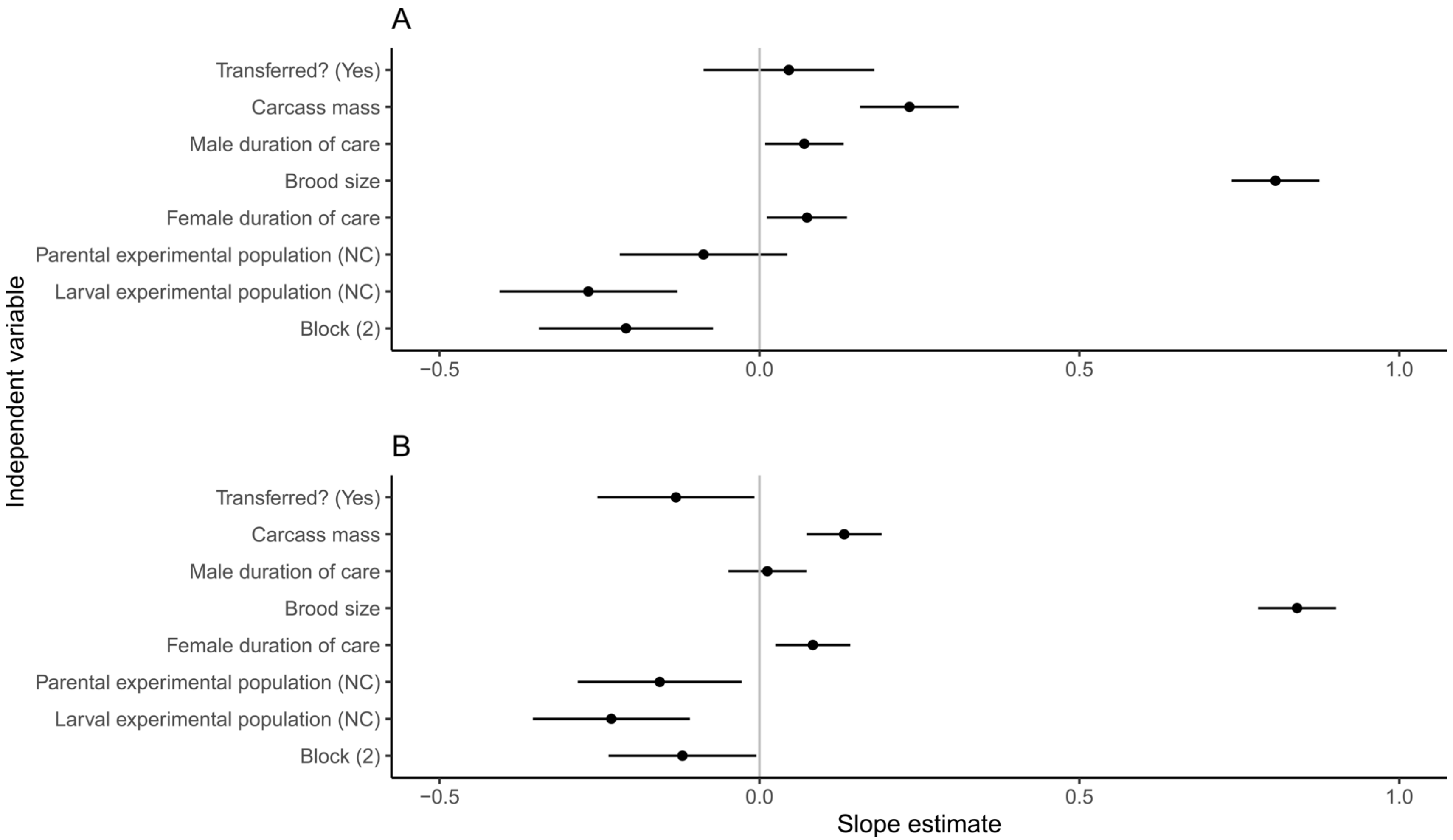

Figure S4 – Coefficient plot showing the value of the standardised regression coefficient (points) with 95% confidence intervals (horizontal lines) for each independent variable predicting brood mass at dispersal at A) generation 24 and B) generation 43. Only those variables that were significant in at least one of the two generations are shown.

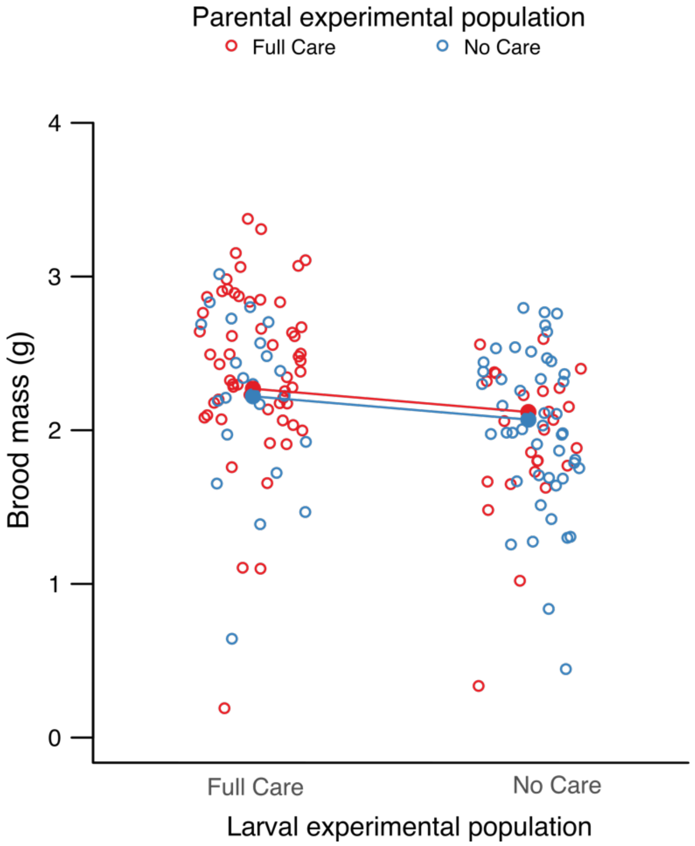

Figure S5 – The effect of larval experimental population of origin, and experimental population of current parents, on brood mass after 23 generations of experimental evolution. Solid points represent the predicted means from the minimal model containing both parental experimental population and larval experimental population terms. Open points represent the actual data points – each point corresponds to an individual brood (n = 153).

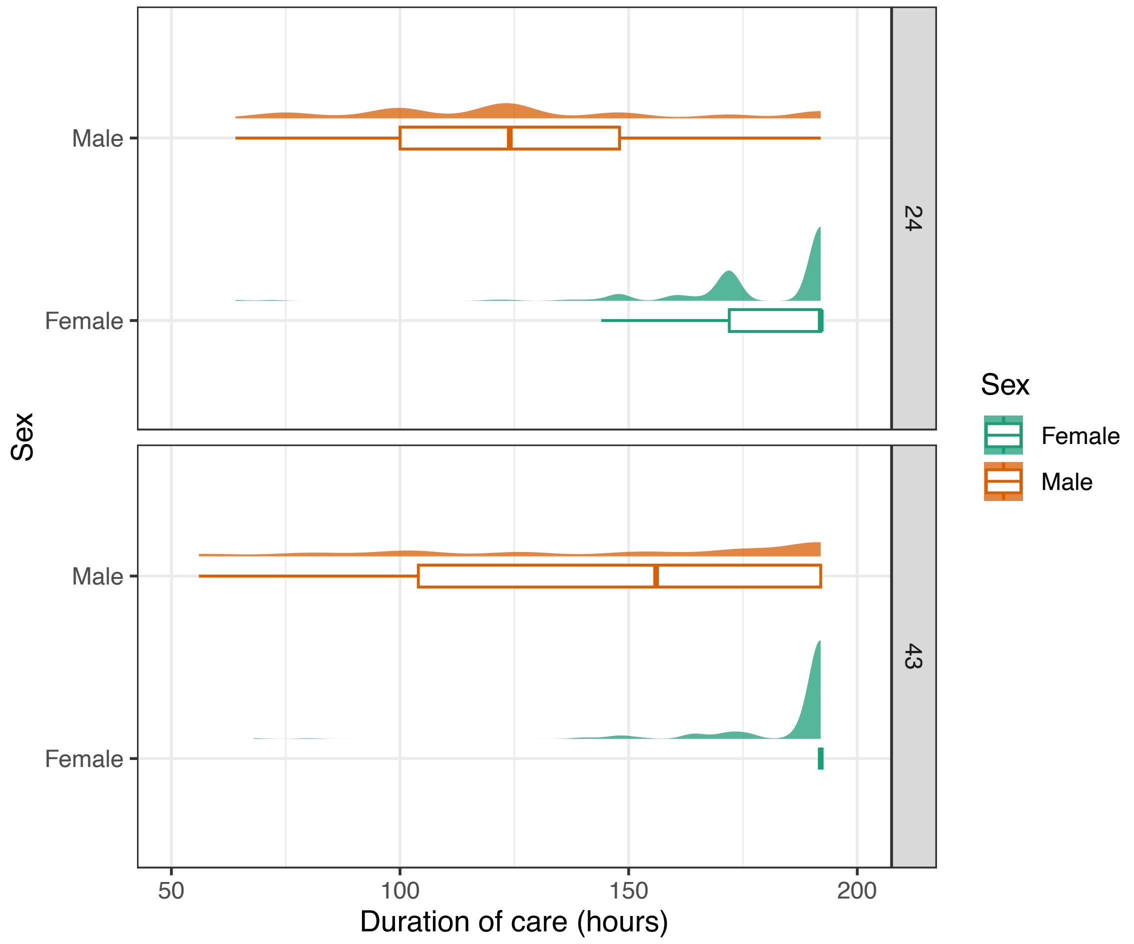

Figure S6 – Duration of care supplied by males and females in generations 24 and 43. Box and density plots showing the duration of maternal (female, n = 369 individuals) and paternal (male, n = 369 individuals) care in generations 24 and 43. Whiskers extend to the farthest data point which is no more than 1.5 times the interquartile range from the box. Density curves show the distributions of individual points.
